# Persistent Catheter-Related *Staphylococcus aureus* Bacteremia after Catheter Removal and Initiation of Antimicrobial Therapy

**DOI:** 10.1371/journal.pone.0046389

**Published:** 2012-10-24

**Authors:** Ki-Ho Park, Yu-Mi Lee, Hyo-Lim Hong, Tark Kim, Hyun Jung Park, So-Youn Park, Song Mi Moon, Yong Pil Chong, Sung-Han Kim, Sang-Oh Lee, Sang-Ho Choi, Jin-Yong Jeong, Mi-Na Kim, Jun Hee Woo, Yang Soo Kim

**Affiliations:** 1 Department of Infectious Diseases, Asan Medical Center, University of Ulsan College of Medicine, Seoul, Republic of Korea; 2 Center for Antimicrobial Resistance and Microbial Genetics, University of Ulsan, Seoul, Republic of Korea; 3 Asan Institute of Life Sciences, Asan Medical Center, University of Ulsan College of Medicine, Seoul, Republic of Korea; 4 Department of Laboratory Medicine, Asan Medical Center, University of Ulsan College of Medicine, Seoul, Republic of Korea; University of Iowa Carver College of Medicine, United States of America

## Abstract

**Objectives:**

Catheter-related *Staphylococcus aureus* bacteremia (CRSAB) occasionally persists despite catheter removal and initiation of appropriate antimicrobial therapy. The aim of this study was to determine the incidence, risk factors, and outcomes of persistent CRSAB after catheter removal and initiation of antimicrobial therapy.

**Methods:**

Consecutive patients with CRSAB were prospectively included from over a 41-month period. We compared the clinical features, 40 bacterial virulence genes, and outcomes between patients with persistent CRSAB (i.e., bacteremia for >3 days after catheter removal and initiation of appropriate antimicrobial therapy) and non-persistent CRSAB.

**Results:**

Among the 220 episodes of CRSAB, the catheter was kept in place in 17 (6%) and removed in 203 (94%) cases. In 43 (21%) of the 203 episodes, bacteremia persisted for >3 days after catheter removal and initiation of antimicrobial therapy. Methicillin resistance (Odds ratio [OR], 9.01; 95% confidence interval [CI], 3.05–26.61; *P*<0.001), non-catheter prosthetic devices (OR, 5.37; 95% CI, 1.62–17.80; *P* = 0.006), and renal failure (OR, 3.23; 95% CI, 1.48–7.08; *P* = 0.003) were independently associated with persistent CRSAB. Patients with persistent CRSAB were more like to experience complication than were those with non-persistent CRSAB (72% vs. 15%; *P*<0.001). Among all episodes due to methicillin-resistant *S. aureus*, persistent CRSAB isolates were associated with accessory gene regulator (*agr*) group II (*P* = .04), but presence of other bacterial virulence genes, distribution of vancomycin minimum inhibitory concentration distribution, and frequency of vancomycin heteroresistance did not differ between the groups.

**Conclusions:**

In patients with CRSAB, bacteremia persisted in 21% of cases despite catheter removal and initiation of antimicrobial therapy. Methicillin resistance, renal failure, and non-catheter prosthetic devices were independent risk factors for persistent CRSAB, which was associated with a higher rate of complications.

## Introduction

*Staphylococcus aureus* bacteremia (SAB) is one of the most common serious bacterial infections worldwide, and intravascular catheters are the most common source of these infections, especially in hospitalized patients [1]. Catheter-related *S. aureus* bacteremia (CRSAB) is a severe healthcare-associated infection that may result in endocarditis, septic thrombophlebitis, metastatic infection, and death [1]–[3]. Bacteremia may persist and complication may develop during the course of therapy if an infected catheter is not removed or if the initiation of antimicrobial therapy is delayed [4], [5]. Thus, catheter removal and initiation of appropriate antimicrobial therapy are essential steps for the optimal treatment of CRSAB.

In practice, however, CRSAB occasionally persists despite catheter removal and initiation of appropriate antimicrobial therapy. There is limited literature evaluating the clinical characteristics and outcomes of patients with persistent CRSAB despite initiation of appropriate therapy. A previous study of 37 patients with CRSAB showed that fever and/or bacteremia that persisted for >3 days after catheter removal and/or initiation of antimicrobial therapy was associated with development of early complications [4]. Furthermore, there is little known about the microbiological and genotypic characteristics of *S. aureus* isolates causing persistent CRSAB. The aim of this study was to determine the incidence, risk factors, and outcomes of persistent CRSAB after catheter removal and initiation of appropriate antimicrobial therapy. We also evaluated the microbiological and genotypic characteristics of isolates associated with persistent CRSAB.

## Methods

### Ethics Statement

Informed consent was waived given that no interventions were planned and collected data were stored anonymously. The Asan Medical Center Institutional Review Board approved the study and waiver of informed consent (IRB number: 2008-0274).

### Study Populations

From August 2008 to December 2011, data were collected as part of a prospective cohort study of *S. aureus* bloodstream infections at Asan Medical Center, Seoul, Korea. During the study period, patients with CRSAB, as defined below, were included. Patients with CRSAB, as defined below, were identified by a daily search of the microbiology laboratory database. Patients younger than 16 years or who had polymicrobial infections were excluded. Only the first episode of bacteremia was included in the analysis to ensure independent observations. During the study period, antibiotic lock therapy and antibiotic-impregnated catheters were not used.

### Definitions

CRSAB was classified as definite or probable according to current IDSA criteria guidelines [6]. CRSAB was considered “definite” (1) if a semiquantitative culture of the removed catheter tip revealed ≥15 colony-forming units by the roll plate technique, and the same organism (by both species and antibiogram) was isolated from the catheter tip and peripheral blood; or (2) indicative differential time to positivity (i.e., the blood culture obtained through catheter became positive at least 2 h earlier than a positive simultaneous blood culture obtained from a peripheral vein) [6], [7]. CRSAB was considered “probable” if the patient had a catheter with at least one positive blood culture for *S. aureus* with compatible clinical presentation and absence of other identifiable source of infection.

CRSAB was considered “persistent” if bacteremia persisted for >3 days after initiation of appropriate therapy. Appropriate therapy was considered to have been initiated if the catheter was removed and if at least one intravenous antibiotic to which the isolate was susceptible was started. CRSAB was considered “non-persistent (1) if bacteremia cleared within 3 days after initiation of appropriate therapy or (2) if follow-up blood cultures were not performed because of resolution of signs and symptoms of the catheter infection after initiation of appropriate therapy.

All surviving patients were followed up 12 weeks after the onset of SAB. Complicated SAB was defined as the presence of (1) attributable mortality, (2) complicated infection present at the time of the initial hospitalization, or (3) late complication. Death was attributable to SAB if blood cultures were positive for *S. aureus* at the time of death or if death occurred before resolution of the signs and symptoms of SAB without another explanation [8]. Complicated infection included infective endocarditis, septic thrombophlebitis, osteomyelitis, septic arthritis, deep tissue abscess, and septic emboli to lungs [2]–[4]. Late complication was defined as the isolation of *S. aureus* from the bloodstream or other sterile body site with the same antibiogram as the initial isolate during the 12-week post-treatment follow-up period [3], [4]. Uncomplicated SAB was defined as no evidence of death due to SAB, complicated infection, or late complication within 12-weeks follow-up period. Designation of death due to a cause other than SAB was based on investigator evaluation during hospitalization and on the death certificate records after discharge.

### Laboratory and Molecular Method

All blood cultures were analyzed using by the BACTEC 9240 (Becton Dickinson, Spark, MD, USA) and all *S. aureus* isolates were identified by standard methods. All blood cultures were analyzed using by the BACTEC 9240 (Becton Dickinson, Spark, MD, USA) and all *S. aureus* isolates were identified by standard methods. Catheter tip cultures were processed by the semiquantitative roll plate culture method [9]. The minimum inhibitory concentration (MIC) of vancomycin was determined using the Etest (AB Biodisk, Solna, Sweden) according to the manufacturer’s instructions. Methicillin-resistant *S. aureus* (MRSA) blood isolates were assessed by the population analysis profiling-area under the curve (PAP-AUC) method, using the technique of Wootton et al [10]. An isolate was identified as hVISA if the ratio of the AUC of the test isolate to the reference strain (Mu3; ATCC 700698) was ≥0.9. The presence of 40 bacterial virulence factors, including adhesins, toxins, *agr* subgroups I–IV, and other genes, were examined by multiplex polymerase chain reaction (PCR), as described elsewhere [11]–[14]. Staphylococcal cassette chromosome (SCC*mec*) types were identified using a previously described method [15].

### Statistical Analysis

Results were analyzed using a commercially available software package (SPSS software, version 14.0 K for Windows; SPSS, Inc., Chicago, IL). Categorical variables were evaluated using the chi-square or Fisher exact test. Continuous variables were compared using the Student *t* test or the Mann-Whitney *U* test, where appropriate. All variables that were significant in univariate analysis were included in a logistic regression model for multivariate analysis. Time to complication was described by the Kaplan-Meier method and compared using the log-rank test. All tests of significance were two-tailed, and a *P* value <0.05 was considered statistically significant.

## Results

### Patients

During the 41-month study period, 239 episodes of CRSAB occurred in 237 adult patients. Two patients had two episodes of CRSAB; only the first episode was included in the analysis. Twelve patients with polymicrobial bacteremia excluded and five patients were lost to follow-up. As a result, 220 patients were included in the analysis. Among them, 135 (61%) were found to have definite CRSAB, and the other 85 (39%) had probable CRSAB.

The source of bacteremia was presumed to be a temporary central venous catheter in 117 (53%), a tunneled cuffed intravascular catheter (e.g., Permcath or Hickman catheter) in 49 (23%), a peripheral vascular catheter in 42 (19%), a peripheral inserted central venous catheter in 5 (2%), a subcutaneous port catheter in 5 (2%), and an arterial catheter in 2 (1%). One hundred and sixty-one patients (73%) had an echocardiogram during the course of therapy; 143 patients (89%) had only transthoracic echocardiogram, and 18 patients (11%) had both transthoracic and transesophageal echocardiogram.

Of the 220 episodes of CRSAB, the catheter was removed from 203 patients (94%), and kept in place in 17 patients (6%). Among the latter 17 patients, 9 recovered, 7 died of SAB, and 1 recovered from SAB but died due to progression of malignancy. Catheter retention group was more likely to have underlying malignancy and long-term intravascular catheters, and to have been received chemotherapy than catheter removal group. Catheter removal group was more likely to be old and to have diabetes mellitus. The complication rate was significantly higher in catheter retained group than catheter removal group (53% [9/17] vs. 27% [55/203], *P* = 0.047) (Table 1).

**Table 1:** Clinical characteristics and outcomes of 220 patients with catheter-related *Staphylococcus aureus* bacteremia according to catheter retention or removal.

Variable	Catheter retained (n = 17)	Catheter removed (n = 203)	*P* value
Age, median (IQR)	50 (46–61)	62 (50–70)	0.02
Male sex	11 (65)	128 (63)	0.89
Community-onset of infection	0 (0)	27 (13)	0.24
Methicillin resistance	9 (53)	122 (60)	0.56
Comorbidity			
Underlying malignancy	14 (82)	108 (53)	0.02
Renal failure	3 (18)	56 (28)	0.57
Diabetes mellitus	0 (0)	56 (28)	0.008
Liver cirrhosis	2 (12)	34 (17)	0.75
Type of catheter			
Central venous catheter	16 (94)	160 (79)	0.21
Long-term intravascular catheters^1^	12 (71)	42 (21)	<0.001
External signs of catheter infection	2 (13)	40 (20)	0.74
Presence of non-catheter prosthetic devices^2^	1 (6)	17 (8)	>0.99
APACHE II score, median (IQR)	18 (15–23)	17 (12–21)	0.38
Pitt bacteremia score, median (IQR)	1 (1–3)	1 (0–3)	0.48
Intensive care unit stay	3 (18)	59 (29)	0.41
Mechanical ventilation	1 (6)	36 (18)	0.32
Prescription of immunosuppressive therapy^3^	5 (29)	43 (21)	0.54
Prescription of cancer chemotherapy^3^	8 (47)	33 (16)	0.005
Recent surgery^3^	2 (12)	57 (28)	0.25
Outcome			
Complicated *S. aureus* bacteremia	9 (53)	55 (27)	0.047
Complicated infection	5 (29)	34 (17)	0.19
Septic thrombophlebitis	1 (6)	19 (9)	>0.99
Infective endocarditis	2 (12)	6 (3)	0.12
Septic emboli to lungs	2 (12)	8 (4)	0.18
Deep tissue abscess	1 (6)	6 (3)	0.44
Septic arthritis	0 (0)	1 (1)	>0.99
Osteomyelitis	0 (0)	1 (1)	>0.99
Attributable mortality	7 (41)	23 (11)	0.003
Late complication	1 (6)	6 (3)	0.44
No complication due to *S. aureus* bacteremia	8 (47)	148 (73)	0.047
Uncomplicated *S. aureus* bacteremia	7 (41)	123 (61)	0.12
Death not-related *S. aureus* bacteremia	1 (6)	25 (12)	0.70

NOTE: Data are no. (%) of patients, unless otherwise indicated. IQR, interquartile range; APACHE II, Acute Physiology and Chronic Health Evaluation II.

1 Includes perm catheter (n = 31), Hickman catheter (n = 18), and subcutaneous port catheters (n = 5).

2 Includes prosthetic valve (n = 7), synthetic vascular graft (n = 6), and orthopedic device (n = 5).

3 Within previous one month.

### Risk Factors Associated with Persistent CRSAB

Of the 203 episodes of CRSAB in which the catheters were removed, bacteremia persisted for >3 days after catheter removal and initiation of appropriate antimicrobial therapy in 43 patients (21%). Clinical characteristics of 203 patients with persistent and non-persistent CRSAB are shown in Table 2. Using univariate analysis, baseline clinical characteristics that were associated with persistent CRSAB included methicillin resistance (*P*<0.001), renal failure (*P*<0.001), central venous catheter (*P* = 0.01), and presence of non-catheter prosthetic devices (*P* = 0.003). Using multivariate analysis, methicillin resistance (odds ratio [OR], 9.01; 95% CI, 3.05–26.61; *P*<0.001), presence of non-catheter prosthetic devices (OR, 5.37; 95% CI, 1.62–17.80; *P* = 0.006), and renal failure (OR, 3.23; 95% CI, 1.48–7.08; *P* = 0.003) were significantly associated with persistent CRSAB. Similar risk estimates were also observed when analyses were restricted to 125 patients with confirmed CRSAB. However, no significant risk factors were observed when analyses were restricted to 78 patients with probable CRSAB (data not shown).

**Table 2:** Clinical characteristics, management, and outcomes of 203 patients with non-persistent and persistent catheter-related *S. aureus* bacteremia after catheter removal and initiation of appropriate antimicrobial therapy.

Variable	Non-persistentCRSAB(n = 160)	PersistentCRSAB(n = 43)	Univariate analysis		Multivariate analysis	
			*P* value	OR (95% CI)	*P* value	OR (95% CI)
Age, median (IQR)	62 (49–70)	64 (53–72)	0.16			
Male sex	101 (63)	27 (63)	0.97			
Community-onset of infection	20 (13)	7 (16)	0.52			
Methicillin resistance	84 (53)	38 (88)	<0.001	6.88(2.57–18.37)	<0.001	9.01(3.05–26.61)
Comorbidity						
Underlying malignancy	86 (54)	22 (51)	0.76			
Renal failure	35 (22)	21 (49)	<0.001	3.41(1.68–6.90)	0.003	3.23(1.48–7.08)
Diabetes mellitus	46 (29)	10 (23)	0.47			
Liver cirrhosis	29 (18)	5 (12)	0.31			
Type of catheter						
Central venous catheter	120 (75)	40 (93)	0.01	4.44(1.30–15.15)		
Long-term intravascular catheters^1^	31 (19)	11 (26)	0.37			
External signs of catheter infection	32 (20)	8 (19)	0.84			
Presence of non-catheter prosthetic devices^2^	8 (5)	9 (21)	0.003	5.03(1.81–13.98)	0.006	5.37(1.62–17.80)
APACHE II score, median (IQR)	17 (12–21)	19 (13–23)	0.13			
Pitt bacteremia score, median (IQR)	1 (0–3)	1 (0–3)	0.76			
Intensive care unit stay	44 (28)	15 (35)	0.34			
Mechanical ventilation	29 (18)	7 (16)	0.78			
Prescription of immunosuppressive therapy^3^	36 (23)	7 (16)	0.38			
Prescription of cancer chemotherapy^3^	28 (18)	5 (12)	0.35			
Recent surgery^3^	45 (28)	12 (28)	0.98			
Clinical management						
Catheter removal within 48 hrs	120 (75)	38 (88)	0.06			
Initiation of appropriate antibiotics within 48 hrs	141 (88)	37 (86)	0.71			
Initial vancomycin use (to MSSA isolates)	31/76 (41)	5/5 (100)	0.02			
Duration of antibiotic therapy	15 (11–21)	27 (20–47)	<0.001			
Outcome						
Complicated *S. aureus* bacteremia	24 (15)	31 (72)	<0.001			
Complicated infection	7 (4)	27 (63)	<0.001			
Septic thrombophlebitis	6 (4)	13 (30)	<0.001			
Infective endocarditis	0 (0)	6 (14)	<0.001			
Other metastatic seeding of infection^4^	1 (1)	15 (35)	<0.001			
Attributable mortality	13 (8)	10 (23)	0.01			
Late complication	4 (3)	2 (5)	0.61			
No complications due to *S. aureus* bacteremia	136 (85)	12 (28)	<0.001			
Uncomplicated *S. aureus* bacteremia	113 (71)	10 (23)	<0.001			
Death not-related *S. aureus* bacteremia	23 (14)	2 (5)	0.09			

NOTE: Data are no. (%) of patients, unless otherwise indicated. CRSAB, catheter-related *Staphylococcus aureus* bacteremia; OR, odds ratio; CI, confidence interval; IQR, interquartile range; MSSA, methicillin-susceptible *S. aureus*; APACHE II, Acute Physiology and Chronic Health Evaluation II.

1 Includes perm catheter (n = 30), Hickman catheter (n = 10), and subcutaneous port catheters (n = 2).

2 Includes prosthetic valve (n = 7), synthetic vascular graft (n = 5), and orthopedic device (n = 5).

3 Within previous one month.

4 Includes septic emboli to lungs (n = 8), deep tissue abscess (n = 6), septic arthritis (n = 1), and osteomyelitis (n = 1).

### Clinical Management and Outcomes of Patients with Persistent CRSAB

Catheter was removed within 48 hrs in 120 patients (75%) with non-persistent CRSAB and in 38 patients (88%) with persistent CRSAB (*P* = 0.06). Appropriate antimicrobial therapy was started within 48 hrs in 141 patients (88%) with non-persistent CRSAB and in 37 patients (86%) with persistent CRSAB (*P* = 0.71). Among patients with bacteremia due to methicillin-susceptible *S. aureus*, all of five patients with persistent bacteremia received vancomycin as the initial antibiotic, but 31 (41%) of 76 patients with non-persistent bacteremia received vancomycin as the initial antibiotic (*P* = 0.02). Patients with persistent CRSAB received more prolonged antibiotic therapy than did those with non-persistent CRSAB (median 27 vs. 15 days; *P*<0.001) (Table 2).

**Figure 1:**
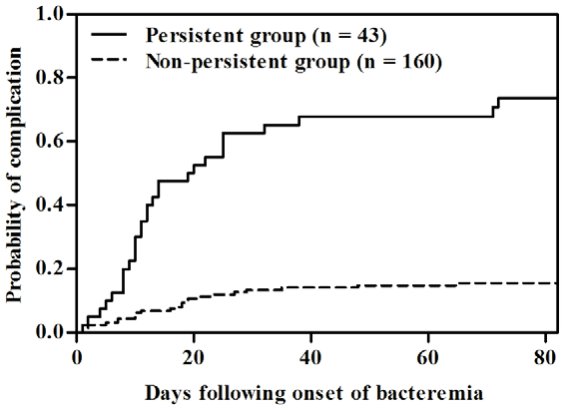
Kaplan-Meier plot showing time to development of complication among patients with catheter-related *Staphylococcus aureus* bacteremia. Complications were more common in patients who had persistent bacteremia for >3 days after catheter removal and initiation of appropriate antimicrobial therapy than in those who did not have persistent bacteremia (log-rank test, *P*<0.001).

Complications occurred in 31 patients (72%) with persistent CRSAB and in 24 patients (15%) with non-persistent CRSAB (*P*<0.001) (Table 2). A Kaplan-Meier plot also showed that the cumulative incidence curves for complications were significantly different between patients with persistent and non-persistent CRSAB (*P*<0.001) (Figure 1). Patients with persistent CRSAB were significantly more like to have complicated infection than those with non-persistent CRSAB (63% [27/43] vs. 4% [7/160], *P*<0.001). Infection-attributable morality was higher in patients with persistent CRSAB than in those with non-persistent CRSAB (23% [10/43] vs. 8% [13/160]; *P* = 0.01). Late complication rates were similar between both groups (5% [2/43] vs. 3% [4/160]; *P* = 0.61) (Table 2). To determine whether persistent CRSAB was independently associated with complications, we performed univariate and multivariate analyses of risk factors associated with the development of complication. Using multivariate analysis, APACHE II score (OR, 1.07; 95% CI, 1.02–1.12; *P*<0.001), persistent status (OR, 13.84; 95% CI, 5.98–32.06; *P*<0.001), and initial inappropriate antimicrobial therapy (OR, 3.04; 95% CI, 1.09–8.45; *P* = 0.03) were independently associated with the development of complications. Among 122 patients infected with MRSA, complication rates were similar according to vancomycin MIC and presence of hVISA phenotype (Table 3).

**Table 3:** Univariate and multivariate analyses of risk factors for development of complication in 203 patients with catheter-related *Staphylococcus aureus* bacteremia.

Variable	No SAB-related complication (n = 148)	SAB-related complication (n = 55)	Univariate analysis		Multivariate analysis	
			*P* value	OR (95% CI)	*P* value	OR (95% CI)
Age, median (IQR)	61 (49–70)	64 (54–71)	0.29	1.01 (0.99–1.04)		
Male sex	91 (62)	37 (67)	0.45	1.29 (0.67–2.48)		
Community-onset of infection	18 (12)	9 (16)	0.43	1.41 (0.59–3.37)		
Methicillin resistance	81 (55)	41 (75)	0.01	2.42 (1.22–4.82)		
Underlying malignancy	74 (50)	34 (62)	0.13	1.62 (0.86–3.05)		
Renal failure	31 (21)	25 (46)	0.001	3.15 (1.62–6.10)		
Diabetes mellitus	42 (28)	14 (26)	0.68	0.86 (0.43–1.74)		
Liver cirrhosis	27 (18)	7 (13)	0.35	0.65 (0.27–1.60)		
Central venous catheter	112 (76)	48 (87)	0.07	2.20 (0.92–5.30)		
Long-term intravascular catheters	28 (19)	14 (26)	0.31	1.46 (0.70–3.05)		
External signs of catheter infection	27 (18)	13 (24)	0.39	1.39 (0.66–2.93)		
Presence of non-catheter prosthetic device	6 (4)	11 (20)	0.001	5.92 (2.07–16.92)	0.052	3.50 (0.99–12.40)
APACHE II score, median (IQR)	16 (11–21)	20 (15–24)	0.001	1.06 (1.02–1.11)	<0.001	1.07 (1.02–1.12)
Pitt bacteremia score, median (IQR)	1 (0–3)	1 (0–4)	0.48	1.03 (0.91–1.18)		
Intensive care unit stay	44 (30)	15 (27)	0.73	0.89 (0.44–1.79)		
Mechanical ventilation	28 (19)	8 (15)	0.47	0.73 (0.31–1.72)		
Prescription of immunosuppressive therapy^1^	29 (20)	14 (26)	0.36	1.40 (0.68–2.91)		
Prescription of cancer chemotherapy^1^	24 (16)	9 (16)	0.98	1.01 (0.45–2.34)		
Recent surgery^1^	41 (28)	16 (29)	0.85	1.07 (0.54–2.12)		
Persistent CRSAB	12 (8)	31 (56)	<0.001	14.64 (6.61–32.42)	<0.001	13.84 (5.98–32.06)
Catheter removal >48 hrs after onset of bacteremia	115 (78)	43 (78)	0.94	1.03 (0.49–2.17)		
Inappropriate antibiotic therapy within 48 hrs	14 (10)	11 (20)	0.04	2.39 (1.01–5.66)	0.03	3.04 (1.09–8.45)
Vancomycin MIC by Etest^2^	(n = 81)	(n = 41)				
≤1.0 mg/L	25 (31)	15 (37)	NA	reference		
1.5 mg/L	38 (47)	19 (46)	0.67	0.83 (0.36–1.94)		
≥2.0 mg/L^3^	18 (22)	7 (17)	0.43	0.65 (0.22–1.91)		
hVISA^2^	27 (33)	18 (44)	0.25	1.57 (0.72–3.38)		

NOTE: Data are no. (%) of patients, unless otherwise indicated. SAB, *Staphylococcus aureus* bacteremia; OR, odds ratio; CI, confidence interval; IQR, interquartile range; Acute Physiology and Chronic Health Evaluation II; CRSAB, catheter-related *S. aureus* bacteremia; MIC, minimum inhibitory concentration; NA, not applicable; hVISA, heteroresistant vancomycin-intermediate *S. aureus*.

1 Within previous one month.

2 Analysis was restricted to 122 MRSA cases.

3 Two isolates had vancomycin MICs of 3 mg/L.

### Microbiological and Genotypic Characteristics of MRSA Isolates Associated with Persistent Catheter-related Bacteremia

Because most episodes (88%) of persistent CRSAB were caused by MRSA, we further evaluated the microbiological and genotypic characteristics of 122 MRSA isolates associated with persistent and non-persistent CRSAB. There were no significant differences in the distribution of vancomycin MIC and frequency of hVISA phenotype. Accessory gene regulator (*agr*) subgroup II was more common in persistent CRSAB isolates than non-persistent CRSAB isolates (90% vs. 73%; *P* = 0.04). Other bacterial virulence genes did not differ between the groups (Table 4).

**Table 4:** Microbiological and genotypic characteristics of 122 methicillin-resistant *Staphylococcus aureus* isolates causing persistent and non-persistent catheter-related bacteremia.

Characteristic	Non-persistent CRSAB (n = 84)	Persistent CRSAB (n = 38)	*P* value
Vancomycin MIC by Etest			0.16
≤1.0 mg/L	23 (27)	17 (45)	
1.5 mg/L	43 (51)	14 (37)	
≥2.0 mg/L^1^	18 (22)	7 (18)	
hVISA	29 (35)	16 (42)	0.42
Adhesin genes			
* clfA*	84 (100)	38 (100)	NA
* clfB*	84 (100)	38 (100)	NA
* cna*	0 (0)	0 (0)	NA
* ebps*	82 (98)	36 (95)	0.59
* fnbA*	84(100)	38 (100)	NA
* fnbB*	82 (98)	38 (100)	>0.99
* map/eap*	3 (4)	2 (5)	0.65
* sdrC*	72 (86)	30 (79)	0.35
* sdrD*	77 (92)	36 (95)	0.72
* sdrE*	81 (96)	36 (95)	0.65
Toxin genes			
* edin*	0 (0)	0 (0)	NA
* Eta*	0 (0)	0 (0)	NA
* Etb*	0 (0)	0 (0)	NA
* Hla*	82 (98)	35 (92)	0.17
* Hlb*	51 (61)	25 (66)	0.59
* Hld*	80 (95)	37 (97)	>0.99
* Hlg*	0 (0)	0 (0)	NA
* hlg-2*	81 (96)	38 (100)	0.55
* lukE-lukD*	81 (96)	38 (100)	0.55
* lukM*	0 (0)	0 (0)	NA
* Pvl*	0 (0)	0 (0)	NA
* Sea*	7 (8)	1 (3)	0.43
* Seb*	0 (0)	0 (0)	NA
* Sec*	56 (67)	31 (82)	0.09
* Sed*	0 (0)	0 (0)	NA
* See*	0 (0)	0 (0)	NA
* Seg*	75 (89)	37 (97)	0.17
* She*	0 (0)	0 (0)	NA
* Sei*	76 (91)	36 (95)	0.72
* Sej*	0 (0)	0 (0)	NA
* Sek*	5 (6)	1 (3)	0.66
* Sel*	66 (79)	34 (90)	0.15
* Sem*	74 (88)	35 (92)	0.75
* Sen*	75 (89)	36 (95)	0.50
* Seo*	75 (89)	37 (97)	0.17
* Sep*	2 (2)	0 (0)	>0.99
* Seq*	5 (6)	1 (3)	0.66
* Tst*	61 (73)	32 (84)	0.16
Other virulence genes			
* icaA*	84 (100)	38 (100)	NA
* agr* subgroup^2^			
* *Subgroup I	21 (26)	4 (10)	0.06
* *Subgroup II	60 (73)	34 (90)	0.04
* *Subgroup III	1 (1)	0 (0)	>0.99
SCCmec type^3^			
* *Type II	63 (76)	34 (89)	0.08
* *Type III	6 (7)	1 (3)	0.43
* *Type IV	14 (17)	3 (8)	0.19

NOTE: Data are no. (%) of isolates, unless otherwise indicated. CRSAB, catheter-related *Staphylococcus aureus* bacteremia; MIC, minimum inhibitory concentration; hVISA, heteroresistant vancomycin-intermediate *S. aureus*; NA, not applicable; SCC*mec*, staphylococcal chromosomal cassette mec.

1 Two isolates had vancomycin MICs of 3 mg/L.

2 Includes 120 isolates: 2 isolates was nontypeable.

3 Includes 121 isolates: 1 isolate was nontypeable.

## Discussion

Optimal management of CRSAB includes early catheter removal and initiation of appropriate antimicrobial therapy [2], [6], [16]. In practice, however, physicians occasionally encounter patients with persistent CRSAB despite catheter removal and initiation of appropriate antimicrobial therapy. We found that 21% of CRSAB cases persisted for ≥3 days after catheter removal and initiation of appropriate antimicrobial therapy. Methicillin resistance, presence of non-catheter prosthetic devices, and renal failure were independently associated with persistent catheter-related bacteremia, which adversely affect patient outcomes.

Persistent bacteremia was more common in episodes caused by MRSA (31% [38/122]) than in those caused by MSSA (6% [5/81]). These results are in line with a prior report documenting the significant association between methicillin resistance and hematogenous complications of CRSAB [2]. One of the possible explanations for these findings may be that glycopeptides are less active against staphylococci than are antistaphylococcal beta-lactams [17]–[20]. In addition, MRSA stains with decreased susceptibility to glycopeptide have emerged, and glycopeptide failure to treat these strains have been reported in various MRSA infection [21]. A recent meta-analysis showed that vancomycin MIC value at the higher end of susceptible range (≥1.5 mg/L) was significantly associated with mortality and treatment failure [22]. Some portion of these failures may be due to unrecognized heterogeneously resistant *S. aureus* (hVISA) which is readily not detected by standard clinical laboratory methods [23]. However, no study stratified MIC data by source of bacteremia, thus it remains unclear whether a high MIC line-related BSI (low risk) has similar implications to a high MIC endovascular BSI (high risk). Among our patients with CRSAB, whose catheters were removed, high vancomycin MIC and hVISA phenotype were not associated with persistent CRSAB and complication. Therefore, our data suggests that when interpreting the impact of MRSA strains with decreased susceptibility on clinical outcomes, outcomes should be stratified by the source of bacteremia or by whether source control was adequate (e.g. intravascular catheter removal).

Because most episodes (88%) with persistent CRSAB were caused by MRSA, we evaluated the microbiological and genotypic characteristics of MRSA isolates associated with persistent CRSAB. Our investigation also showed that *agr* group II was associated with persistent CRSAB. Previously, *agr* group II was linked to vancomycin failure in one study [24], but not in another study [20]. We evaluated several bacterial virulence factors, including adhesin and toxin genes, but we could not find any association between these virulence genes and persistent CRSAB.

The presence of non-catheter prosthetic devices was an independent risk factor for persistent CRSAB in this study. This observation is consistent with prior reports documenting high rates of seeding by *S. aureus* in a variety of noncatheter prosthetic devices [25]–[29]. Noncatheter prosthetic devices can serve as a focus for hematogenous spread of SAB. The current investigation also demonstrated that renal failure was an independent risk factor for persistent CRSAB. This finding is consistent with the report by Fowler et al. who found that renal failure was an independent risk factor for hematogenous complications [2]. A more recent study by Ghanem et al. found that renal failure at the onset of CRSAB was associated with a high risk of early complications [3]. This elevated risk may be related to uremia-associated phagocytic dysfunction [30]. Renal failure may contribute to complication or persistent bacteremia by superantigen-dependent enhancement of endotoxin shock and renal tubular cell injury [31], [32].

Persistent bacteremia after catheter removal and initiation of appropriate antimicrobial therapy usually reflects serious complications of CRSAB, such as septic thrombophlebitis, endocarditis, or metastatic foci of infection [4], [33]. A previous report showed that acute early complications of CRSAB more frequently occurred during the initial course of therapy in patients in which fever and/or bacteremia persisted for >3 days after catheter removal than in those who responded within 3 days after catheter removal (7/8 [88%] vs. 1/29 [3%]; *P*<0.001) [4]. Our study included a large number of cases of CRSAB that were prospectively followed; we confirmed the high rate of early complications in patients with persistent CRSAB compared with non-persistent CRSAB.

The current study has several limitations. First, it was conducted in a single tertiary care institution and referral center. This may have caused a selection bias towards more severe or complicated cases, resulting in limitations on the generation of the results. Second, clearance of bacteremia was not documented for some patients with non-persistent CRSAB in whom clinical symptoms and signs of CRSAB frequently resolved after catheter removal and initiation of appropriate antimicrobial therapy. However, similar observations were made after analysis was restricted to patients in whom clearance of bacteremia was documented (data not shown). Third, we did not use PFGE to type MRSA blood isolates, and we thus could not evaluate the possibility that clonal relationships may exist among some isolates.

### Conclusion

Bacteremia persisted for >3 days after catheter removal and initiation of appropriate antimicrobial therapy in 21% of our CRSAB cases. Baseline risk factors for persistent CRSAB were methicillin resistance, presence of non-catheter prosthetic devices, and renal failure. Persistent CRSAB was associated with high rates of acute complications and infection-related mortality, and its optimal management remains challenging for clinicians.
